# Circulating Interleukin-4 Is Associated with a Systemic T Cell Response against Tumor-Associated Antigens in Treatment-Naïve Patients with Resectable Non-Small-Cell Lung Cancer

**DOI:** 10.3390/cancers12123496

**Published:** 2020-11-24

**Authors:** Seyer Safi, Yoshikane Yamauchi, Hans Hoffmann, Wilko Weichert, Philipp J. Jost, Hauke Winter, Thomas Muley, Philipp Beckhove

**Affiliations:** 1 Division of Thoracic Surgery, University Hospital Rechts der Isar, Technical University of Munich, Ismaninger Strasse 22, 81675 Munich, Germany; hans.hoffmann@mri.tum.de; 2 Department of Surgery, Teikyo University School of Medicine, Tokyo 173-8605, Japan; yoshikaney@med.teikyo-u.ac.jp; 3 Institute of Pathology, Technical University of Munich, Trogerstrasse 18, 81675 Munich, Germany; wilko.weichert@tum.de; 4 Medical Department III for Hematology and Oncology, University Hospital Rechts der Isar, Technical University of Munich, Ismaninger Strasse 22, 81675 Munich, Germany; philipp.jost@tum.de; 5 German Consortium for Translational Cancer Research (DKTK) of the German Cancer Research Center (DKFZ), 69120 Heidelberg, Germany; 6 Division of Clinical Oncology, Department of Medicine, Medical University of Graz, Auenbruggerplatz 15, 8036 Graz, Austria; 7 Department of Thoracic Surgery, Thoraxklinik, Heidelberg University Hospital, Roentgenstrasse 1, 69126 Heidelberg, Germany; hauke.winter@med.uni-heidelberg.de; 8 Translational Lung Research Center (TLRC), Member of German Center for Lung Research (DZL), 69120 Heidelberg, Germany; thomas.muley@med.uni-heidelberg.de; 9 Translational Research Unit, Thoraxklinik, Heidelberg University Hospital, Roentgenstrasse 1, 69126 Heidelberg, Germany; 10 Regensburg Center for Interventional Immunology and Regensburg University Hospital, Franz-Josef-Strauss-Allee 11, 93053 Regensburg, Germany; beckhove@rcii.de

**Keywords:** lung cancer, cytokine, T cells, immunotherapy

## Abstract

**Simple Summary:**

Cytokines can increase the activity of T cells specific for tumor-associated antigens and thereby promote tumor-specific immune responses. In this study, cytokine profiles and T cell responses against 14 tumor-associated antigens were investigated in 36 treatment-naïve patients with resectable non-small cell lung cancer. Based on these results, preoperative serum interleukin-4 levels can play a role in predicting T cell responses specific for tumor-associated antigens and recurrence-free survival regardless of tumor stage. This is clinically relevant as patients with high preoperative serum interleukin-4 levels could be at high risk of postoperative tumor recurrence and, therefore, should be considered for adjuvant or neoadjuvant treatment. From this perspective, preoperative serum interleukin-4 levels may become a useful option to assess the risk of postoperative tumor recurrence in non-small-cell lung cancer.

**Abstract:**

Spontaneous T cell responses to tumor-associated antigens (TAs) in the peripheral blood of patients with non-small-cell lung cancer (NSCLC) may be relevant for postoperative survival. However, the conditions underlying these T cell responses remain unclear. We quantified the levels of 27 cytokines in the peripheral blood and tumor tissues from treatment-naïve patients with NSCLC (*n* = 36) and analyzed associations between local and systemic cytokine profiles and both TA-specific T cell responses and clinical parameters. We defined T cell responders as patients with circulating T cells that were reactive to TAs and T cell nonresponders as patients without detectable TA-specific T cells. TA-specific T cell responses were correlated with serum cytokine levels, particularly the levels of interleukin(IL)-4 and granulocyte colony-stimulating factor (G-CSF), but poorly correlated with the cytokine levels in tumor tissues. Nonresponders showed significantly higher serum IL-4 levels than responders (*p* = 0.03); the predicted probability of being a responder was higher for individuals with low serum IL-4 levels. In multivariable Cox regression analyses, in addition to IL-4 (hazard ratio (HR) 2.8 (95% confidence interval (CI): 0.78–9.9); *p* = 0.116), the age-adjusted IL-8 level (HR 3.9 (95% CI: 1.05–14.5); *p* = 0.042) predicted tumor recurrence. However, this study included data for many cytokines without adjustment for multiple testing; thus, the observed differences in IL-4 or IL-8 levels might be incidental findings. Therefore, additional studies are necessary to confirm these results.

## 1. Introduction

Non-small-cell lung cancer (NSCLC) constitutes approximately 85% of all new lung cancer cases [1] and is the leading cause of cancer-related death [2]. Following complete resection and adjuvant chemotherapy, the 5-year survival rate of patients with NSCLC is only approximately 60% [3,4]. T cell-based cancer immunotherapy provides new therapeutic options for the management of NSCLC. Spontaneous T cell responses are now recognized as a prognostic factor for patients with lung cancer [5]. As shown in our previous study, the peripheral blood of patients with NSCLC harbors tumor antigen (TA)-specific T cells, and the presence of these functionally active and circulating T cells correlates with improved recurrence-free survival (RFS) [6].

Cytokines are soluble molecular messengers. Immunoregulatory cytokines not only play a crucial role in the initiation and promotion of tumors [7] but also represent an essential component of signaling pathways during cancer-related immune responses [8]. The expression of cytokines in patients with cancer is interpreted as a paraneoplastic phenomenon based on tumor-induced immune stimulation, with only local immunosuppression occurring in early tumor stages and systemic immunosuppression observe in later tumor stages; this immunosuppression protects cancer cells from immunological eradication [9]. An association between the concentrations of circulating cytokines and tumor prognosis has also been reported in patients with NSCLC [9]. Despite all the work published to date, researchers have not clearly determined how and to what extent the spontaneous generation of circulating TA-specific T cells is associated with local and systemic cytokine signatures. To date, several studies have investigated the cytokine profile only in the peripheral blood of patients with advanced stages of NSCLC [10,11,12,13,14,15], but data from patients with early-stage disease and tumor cytokine profiles are largely lacking. In addition, the simultaneous measurement of different cytokines is important for the investigation of interactions between cytokines, but has been reported only infrequently. The simultaneous functions of many cytokines in both immune activation and immune suppression poses a major challenge for achieving effective antitumor reactions without causing treatment-limiting toxicity. Here, we performed a comprehensive analysis of 27 inflammatory cytokines in representative tumors and corresponding serum samples from patients with untreated NSCLC who were undergoing surgery with a curative intent. Data on circulating T cells directed against 14 NSCLC-associated antigens were correlated with the local intratumor and systemic cytokine profiles. The goal of this study was first to preoperatively identify TA-specific T cell responders based on the cytokine levels and preoperative information and second to preoperatively identify the patient group at high risk of postoperative tumor recurrence based on measurements of cytokine levels or TA-specific T cell responses. The primary objective of this study was to identify local and systemic cytokine signatures that are associated with the presence of a spontaneous TA-specific immune response in treatment-naïve patients with resectable NSCLC.

## 2. Results

We defined T cell responders as patients with circulating T cells that were reactive to any of the 14 tested TAs and T cell nonresponders as patients without detectable TA-specific T cells. Enzyme-linked immunospot (ELISPOT) data for TA-specific immune responses in peripheral blood and corresponding Bioplex data for cytokine levels in serum and tumor tissues were available for 36 patients (Figure S1). The current study (*n* = 36) is an analysis of a subgroup of our previously published study population (*n* = 51) [6]. The characteristics of the patients analyzed in the current study are shown in Table S1.

### 2.1. Association of the TA-Specific Response with Clinical Parameters

The average age of nonresponders (*n* = 14) was 70 years compared to an average age of 63.9 years for responders (*n* = 22, *p* = 0.059, *t*-test). As reported previously [6], the interferon(IFN)-γ spot counts correlated significantly with RFS (*n* = 36; Figure S2a,b). No association was observed between the response groups (responder versus nonresponder) and other prognostic factors, such as histologically assessed lymphangiosis carcinomatosa, adjuvant chemotherapy, lymph node metastases and sex (Table S1). In summary, we identified more nonresponders among the older patients and more responders among the younger patients. However, significant differences in sex, smoking behavior, surgical resection extent, tumor size, tumor histology, postoperative International Union against Cancer (UICC) tumor stage, lymphangiosis carcinomatosa, or lymph node metastasis were not observed between older and younger patients (Table S2).

### 2.2. Association of the TA-Specific Response with Intratumor and Serum Cytokine Levels

**Figure 1:**
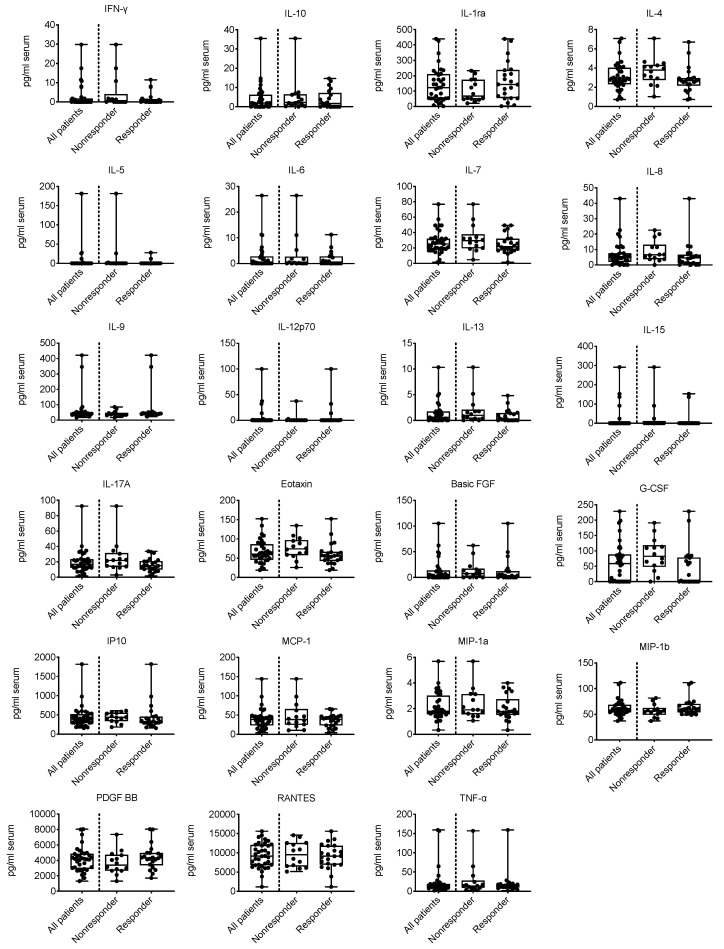
Box and whisker plots representing the preoperative serum cytokine levels in all patients (*n* = 36), responders only (*n* = 22) and nonresponders only (*n* = 14). FGF = fibroblast growth factor. G-CSF = granulocyte colony-stimulating factor. IFN = interferon. IL = interleukin. IP10 = interferon-inducible protein 10. MCP-1 = monocyte chemoattractant protein 1. MIP = macrophage-inflammatory protein. PDGF = platelet-derived growth factor. RANTES = regulated upon activation, normal T cell expressed and secreted. TNF = tumor necrosis factor.

The Bioplex data for measurements of cytokine levels in serum and tumor tissues are summarized in Tables S3 and S4. Table 1 presents estimates of the correlations between the serum cytokine levels and TA-specific responses. Corresponding box and whiskers plots were prepared to visualize the distribution of the data (Figure 1 for serum cytokine levels and Figure S3 for tumor cytokine levels). The highest absolute correlations were observed for granulocyte colony-stimulating factor (G-CSF), followed by interleukin(IL)-4 and eotaxin. We detected significantly higher median serum G-CSF and IL-4 levels in nonresponders (Wilcoxon–Mann–Whitney test). In contrast to the serum G-CSF and IL-4 levels, a tendency toward a positive correlation between cytokine levels and TA-specific responses was observed for serum IL-9 levels (*p* = 0.091). The association between serum IL-4 levels and TA-specific responses was shown not only for TA-specific responses dichotomized into responders and nonresponders but also between IL-4 levels and the strength of the TA-specific response, as indicated by the median number of IFN-γ spots minus the negative control (Table S5 and Figure S4). Since our analyses only revealed a correlation between TA-specific responses and serum cytokine levels and not between TA-specific responses and the cytokine levels in the tumor tissues (Table S6), we limited our further investigations to the cytokine levels in serum and excluded the cytokine levels in tumor tissues.

Thus, the presence of circulating TA-specific T cells, as detected in ELISPOT assays, was correlated with cytokine levels in the serum, but not with cytokine levels in the tumor tissues. Nonresponders showed significantly higher serum IL-4 levels.

### 2.3. Identification of Distinct Clusters

**Figure 2:**
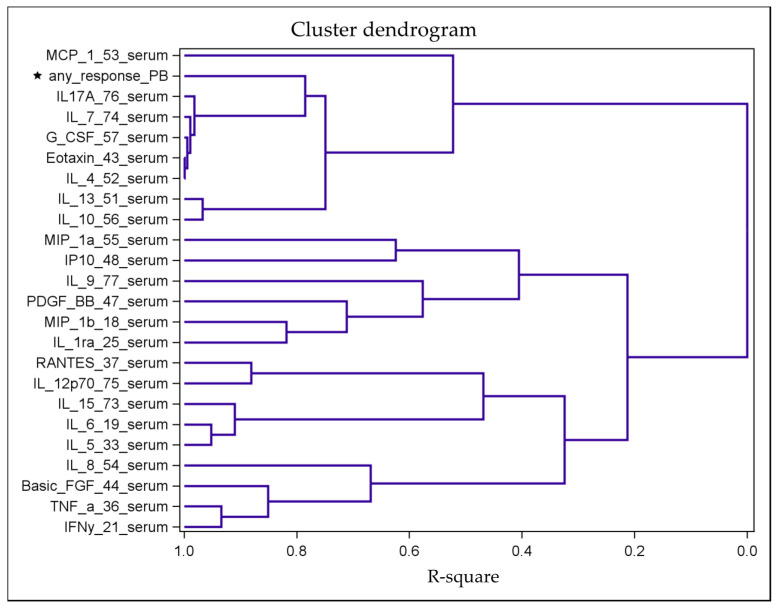
Tree-structured dendrogram resulting from the hierarchical clustering analysis based on the correlation matrix of all cytokine and response data. The R-square value is the proportion of variance accounted for by the cluster. For example, the variables IL-4, eotaxin, G-CSF, IL-7 and IL-17A represent a cluster of variables with similar correlations with the response variable (“any_response_PB”; marked with an asterisk). In contrast, the dendrogram shows a marked distance between the variables IL-6 and IL-8 and the response variable, indicating a weak correlation.

As cytokines interfere directly or indirectly with the expression of other cytokines, the isolated effect of one cytokine may appear less relevant unless it is assessed in the context of a defined cascade [9]. We performed a hierarchical clustering analysis based on the correlation matrix of all variables as a proximity measure to visualize groups of interacting cytokines that modulate TA-specific responses. As a result, a tree-structured dendrogram was established (Figure 2).

We found first that the serum variables IL-4, eotaxin, G-CSF, IL-7, and IL-17A built a cluster of similar correlation structures, and second, that this cluster of cytokines already formed a cluster with the response variable at the next level. The correlations between these variables (IL-4, IL-7, IL-17A, eotaxin, and G-CSF) were very strong (ranging from 0.70 to 0.95; Figure S5). Regarding the following multivariable models, this collinearity indicated that only one of these five cytokine variables in the cluster was considered an independent variable for the models, while the variable TA-specific response remained the dependent variable. Therefore, in addition to serum IL-4 levels, the serum levels of IL-7, IL-17A, eotaxin, and G-CSF show not only similar correlations to the response variable but also strong correlations among each other.

### 2.4. Modeling the Probability of Being a Responder

**Figure 3:**
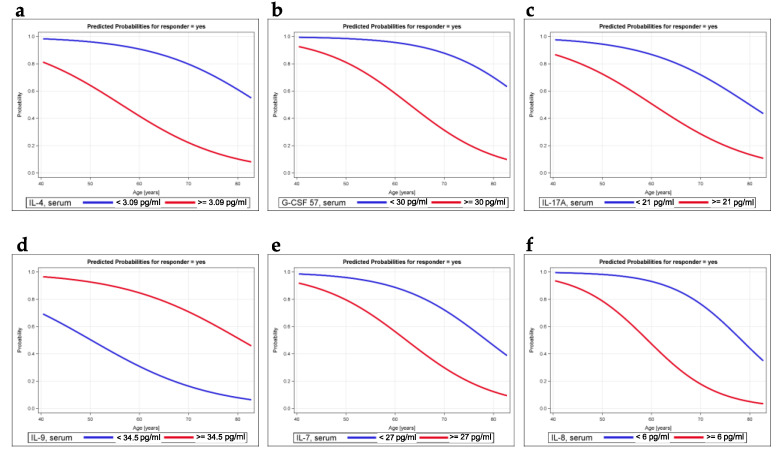
Predicted probability of a response obtained from age-adjusted logistic models. As an example, the age-weighted probability of being a responder based on cytokine levels is shown for the serum cytokines IL-4 (**a**), G-CSF (**b**), IL-17A (**c**), IL-9 (**d**), IL-7 (**e**), and IL-8 (**f**). The blue curves illustrate the probability of having serum cytokine levels below the optimal cutoff, and the red lines represent the probability of having serum cytokine levels above the cutoff.

Next, we investigated the diagnostic value of serum cytokine patterns for determining whether a patient had circulating TA-specific T cells. Therefore, we modeled the probability of being a responder based on cytokine profiles. Further analyses were restricted to those variables with a tendency toward an association, i.e., *p* < 0.2 based on the Wilcoxon–Mann–Whitney test or a Spearman correlation coefficient >0.23 in Table 1. The results of crude and age-adjusted models are shown in Table 2 for the optimal cutoffs of serum cytokine levels. When the variables were defined as continuous variables, the correlation between TA-specific responses and cytokine levels was less pronounced. The adjustment for age strengthened the positive effects and reduced the negative effects. Significant associations were identified for IL-4, G-CSF, eotaxin, IL-7, and IL-17A, as well as for IL-9 and IL-8 (Table 2). The predicted probabilities of being a responder are illustrated in Figure 3.

Thus, with increasing age, the probability of being a responder decreased over time and was generally lower in patients with serum cytokine levels below the optimal cutoff values, with the exception of serum IL-9 levels; higher values were more frequently detected in responders than in nonresponders.

### 2.5. Multivariable Model for Being a Responder

**Figure 4:**
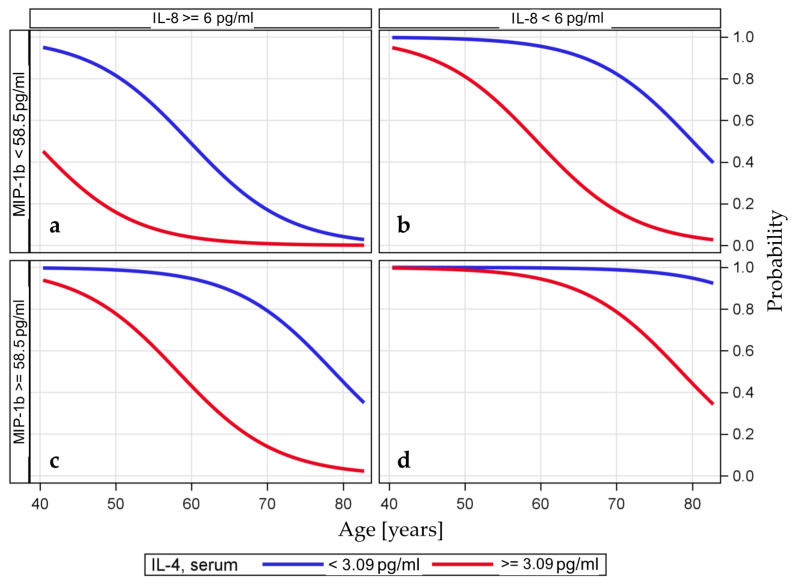
Predicted probability of a TA-specific response based on a multiple logistic model including the variables IL-4, IL-8, and MIP-1b (dichotomized at the optimal cutoff value) and age (continuous variable). The predicted probability of being a responder is visualized based on the three serum cytokines dichotomized at their optimal cutoff values and the variable age. The probabilities of having IL-4 levels above the optimal cutoff value are shown as blue lines, and the probabilities of having IL-4 levels below the cutoff value are shown as red lines. The categorization according to the optimal cutoff value of MIP-1b is shown in the upper and lower rows and the categorization for IL-8 is shown in the left and right columns. The variable age is used as a continuous variable on the *x*-axis. For example, the probability of being a responder is very high if the serum IL-4 level is below the threshold, the serum IL-8 level is also below the threshold, and the serum MIP-1b level is above the threshold (**d**). In contrast, the likelihood of being a responder in the older age group is very low if the serum IL-8 level is above the threshold and the serum MIP-1b level is also below the threshold (**a**). Likelihood of being a responder if the serum IL-8 level is below the threshold and the serum MIP-1b level is also below the threshold (**b**). Likelihood of being a responder if the serum IL-8 level is above the threshold and the serum MIP-1b level is also above the threshold (**c**).

We developed a multivariable model using the forward selection approach starting with all serum cytokine variables with bivariate associations at a level of *p* < 0.15 (Table 2) and with the potential risk factors for tumor recurrence (Table S7). None of the risk factors for tumor recurrence met the criteria for inclusion. The most significant variable was IL-4. The subsequent stepwise selection led to the generation of a multivariable model for the TA-specific response, with statistically significant effects observed for age, IL-4, IL-8, and MIP-1b; high odds ratios and large confidence intervals were obtained due to the small numbers used for estimation (Table 3). Figure 4 illustrates the predicted probability of being a responder based on the serum levels of the cytokines IL-4, IL-8, and MIP-1b and the variable age.

The multivariable model confirmed first that the predicted probability of being a responder might be greater for individuals with low serum IL-4 levels than for patients with high serum IL-4 levels, and second, the probability of being a responder may decrease with increasing age. The variables MIP-1b and IL-8 modified the probability of being a responder, as the effect of age on the probability of being a responder for patients with low serum MIP-1b levels and high serum IL-8 levels was observed very early, while the effect of age was observed later for patients with high serum MIP-1b levels and low serum IL-8 levels (Figure 4).

### 2.6. Associations between Cytokine Levels and Risk Factors for Tumor Recurrence

We next examined the associations between dichotomized serum cytokine levels and established risk factors or confounders for tumor recurrence following the complete resection of NSCLC with curative intent (Table S7). Differences in serum IL-4, G-CSF, and eotaxin levels were observed in patients with confirmed lymphangiosis carcinomatosa (*p* < 0.10). For example, in patients without lymphangiosis carcinomatosa (pL0), 25% of serum IL-4 levels were above the cutoff, while in patients with confirmed lymphangiosis carcinomatosa (pL1), up to 60% of serum IL-4 levels were above the cutoff. Therefore, the serum levels of IL-4, G-CSF and eotaxin, which were associated with TA-specific responses, were also associated with lymphangiosis carcinomatosa.

### 2.7. Association of Cytokine Levels with RFS

**Figure 5:**
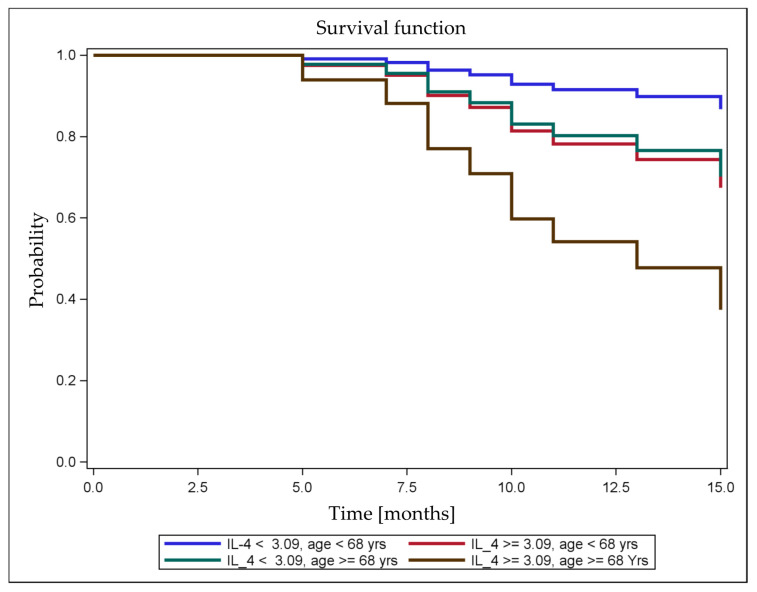
Postoperative survival curves predicted using the Cox model. Postoperative RFS after curative-intent surgery for NSCLC was predicted based on the variables serum IL-4 levels and age. Prolonged survival was predicted for younger patients with low serum IL-4 levels than for older patients with high serum IL-4 levels. The favorable effect of younger age on survival was reversed by the combination with higher serum IL-4 levels.

As shown in our previous study of patients with completely resected NSCLC, the presence of circulating TA-specific T cells was associated with prolonged RFS [6]. Here, we developed crude and age-adjusted Cox regression models for postoperative RFS using the serum cytokine levels and observed a relationship with TA-specific T cell responses. We found the strongest effects for IL-4, G-CSF, and IL-8 on postoperative RFS. With *p*-values < 0.15, the serum cytokine levels showed only a trend toward an effect on RFS (Table 4). Only IL-8 showed a difference in the effect on RFS after adjustment for age, with a change in HR from 2.7 in the crude Cox regression to an age-adjusted HR of 3.9 (Table 4). The predicted postoperative survival curves based on the Cox model for serum IL-4 levels and age (Figure 5) revealed independent effects: both a younger age and lower IL-4 levels were associated with a lower risk of postoperative tumor recurrence. In addition, older patients with low IL-4 levels had a risk similar to younger patients with high IL-4 levels. In this case, the effects of age and IL-4 cytokine levels may cancel each other out. Due to the significant correlation between TA-specific T cell responses and IL-4 levels, the effect of IL-4 levels on RFS disappeared when the TA-specific response was included in the model as a predictor variable in addition to IL-4 levels.

In addition to the predicted survival, the association between the preoperative serum IL-4 level and the observed postoperative RFS is presented in Figure S6.

The postoperative tumor stage (Figure S6a), lymph node metastases (Figure S6b), and lymphangiosis carcinomatosa (Figure S6c) were decisive factors for RFS. For lymphangiosis carcinomatosa, we already showed an association with serum IL-4 and G-CSF levels (Table S7). Other multivariable models including the factor lymphangiosis carcinomatosa did not provide further insights, as lymphangiosis carcinomatosa proved to be such a strongly influential factor that the importance of the other factors in the model was diminished.

## 3. Discussion

The most important finding of this study was the possible role of the serum level of the cytokine IL-4 as a circulating biomarker for TA-specific T cell responses in treatment-naïve patients with resectable NSCLC (*n* = 36; Figure S1).

ELISPOT assays are effective tools to analyze the function of circulating TA-specific T cells and evaluate immunological outcomes; thus, they are already used in vaccination studies. The disadvantage is that ELISPOT assays are still time-consuming and costly, which is why further development of the current ELISPOT method is underway [16]. In this context, the use of circulating cytokines as a surrogate biomarker may allow researchers to draw conclusions about cellular immune responses by analyzing a blood sample with a comparably simple and cheap method.

First, we investigated the associations between TA-specific T cell responses in the blood and clinical parameters. We identified more nonresponders among the older patients and more responders among the younger patients (*p* = 0.059; Table S1). The observed effect of age may be due to immunosenescence, which is a process that is considered to cause a decrease in general immune function in the elderly. Significant decreases in cytotoxic activity during the aging process have been described for effector immune cells, including T cells [17]. Moreover, many studies have shown significantly increased numbers of immunosuppressive myeloid suppressor cells (MDSCs) [18] and regulatory T cells [19] in the tissues and blood of elderly patients; both of these cell types inhibit tumor-specific T cell functions and lead to tumor progression [20,21]. The age-related increase in the number of responders among younger patients observed in this study might potentially be explained by the age-related decrease in the functional activity of TA-specific T cells. However, to the best of our knowledge, a direct relationship between age and TA-specific T cell responses has not yet been documented.

Next, we investigated the relationship between TA-specific responses and both circulating blood and intratumor cytokine levels. We detected significantly higher serum IL-4 levels in nonresponders (*p* = 0.030, Table 1). This finding appears to be consistent with a report from Wijesundara et al. [22] showing that IL-4 regulated the quality of the immune response during viral infection in a murine model in such a way that the protective CD8^+^ T cell response was impaired. Additionally, in a murine model, Kienzle et al. [23] described the ability of IL-4 to downregulate the cytotoxic function and the expression of IFN-γ in CD8^+^ T cells during primary activation.

With few exceptions, the cytokine levels measured in this study in serum and tumor samples were similar to the values reported in the literature [24,25,26,27] or in previous publications from our group [28]. Cytokines exert their biological effects at very small doses and have short half-lives. The variability in the measured cytokine levels is also increased by the presence of secondary diseases in the examined patients, such as chronic inflammation, e.g., chronic obstructive pulmonary disease (COPD), or aging and preanalytical factors, such as fasting, medication, physical activity, blood processing, and analytical factors [29]. For cytokine concentrations to be compared between different measurements, they must be reported in a standardized manner, for example, as pg of cytokines per mg of total protein content. However, this latter condition is not always met, and comparisons of cytokine levels in the blood with cytokine levels in tumor tissue or comparisons of cytokine levels between different studies are limited.

Cancer cells are protected from immunological eradication by cytokine-mediated local immunosuppression. In particular, the cytokines in the tumor tissue, which are produced by the tumor itself, are linked to this mechanism [9]. On the one hand, we were surprised that the TA-specific T cell response was correlated with the serum cytokine levels but not with the cytokine levels in tumor tissues. On the other hand, in a previous study of patients with resectable NSCLC, we were able to document a link between postoperative disease progression and circulating MDSCs and the levels of cytokines produced by these cells in the blood, but not with MDSCs in the tumor tissue [28]. Conclusions about cytokines in the tumor tissue and TA-specific responses must be drawn with caution, because only a few studies have reported cytokine levels in tumor tissue, and the majority of previous studies have concentrated on cytokine levels in the serum. Moreover, tumors have a heterogeneous composition and thus intratumorally measured cytokine levels show high variability, depending on the location of sampling within the tumor tissue. In the present study, cytokine levels were only measured in tumor samples for which the microscopic proportion of tumor cells in the sample was considered by a pathologist to be at least 40% to minimize the effect of this latter confounding factor. Nevertheless, we have excluded the cytokine data obtained from tumor tissues from further analyses, because a clear association between the cytokine levels in the tumor tissue and a TA-specific T cell response was not observed in this patient population.

In addition to serum IL-4 levels, the serum levels of IL-7, IL-17A, eotaxin, and G-CSF not only showed a similar negative correlation with the TA-specific response but also strong correlations among each other. Despite the known partially opposing functions of cytokines, these five cytokines are commonly associated with tumor initiation or progression in different types of tumors [30,31,32,33].

We addressed the question of whether a specific cytokine pattern is potentially useful to estimate the presence of a TA-specific T cell response and developed age-adjusted logistic regression models. The probability of being a responder was most strongly associated with the serum IL-4 level (Table 2). For example, according to this adjusted model, the probability of being a responder was 13.8 times higher in patients with serum IL-4 levels lower than 3.09 pg/mL than in patients with serum IL-4 levels of at least 3.09 pg/mL (Table 2), which is a correlation that was also confirmed in the multivariable model, where we also observed statistically significant effects of age, IL-8, and MIP-1b (Table 3). The decreasing probability of being a responder with aging (Figure 3) confirms our observed association between age and the presence of a TA-specific response (Table S1).

IL-4 was associated not only with the dichotomized form of the TA-specific T cell response (yes versus no; see Section 4.8 *IFN-γ ELISPOT assays*) but also with the median IFN-γ spot counts (Table S5). Unlike other cytokines, our analyses repeatedly indicated a role for IL-4 in the TA-specific response. IL-4 is a multifunctional cytokine that is predominantly produced by T helper 2 cells and most likely also by MDSCs [34] and regulates immune responses and the immune microenvironment [35]. The number of IL-4-producing T cells is increased in patients with NSCLC, which is associated with a worse prognosis [36]. After the administration of a neutralizing antibody against IL-4 in a murine model of breast and colon cancer, increased antitumor immunity and delayed tumor progression were reported. IL-4 blockade not only reduced the formation of immunosuppressive M2 macrophages and MDSCs but also increased the function of tumor-specific cytotoxic T cells [37]. In an additional study of an autologous approach using tumor samples and peripheral blood mononuclear cells from patients with colorectal cancer, the blockade of IL-4 was associated with an increased efficiency of tumor-specific T cell reactivity [38].

An association between the concentrations of circulating cytokines and the tumor prognosis has also been reported for other cytokines in patients with NSCLC. In a case-control study from the National Cancer Institute, Maryland, Pine et al. [10] showed a prognostic association between increased serum IL-8 levels several years before diagnosis and a higher lung cancer risk. The predictive value of serum IL-8 levels as a biomarker for the response to programmed cell death protein-1 (PD-1) blockade was evaluated by Sanmamed et al. [11] in an immunotherapy study that included 29 patients with melanoma or NSCLC. In patients who responded to PD-1 blockade, serum IL-8 levels were significantly decreased from the baseline to the time of best response and significantly increased upon tumor progression [10]. In an interventional clinical study including patients with NSCLC, the administration of recombinant human IL-15 was associated with increases in the numbers of circulating natural killer and CD8^+^ memory T cells [12,13]. Therefore, combination therapies that include cytokines are being investigated to increase the low response rate to immunotherapies [39]. In a recent phase 1 trial in patients with metastatic NSCLC, the subcutaneous addition of a recombinant IL-15 agonist to the PD-1 monoclonal antibody nivolumab induced Ki-67 expression in CD8^+^ T and natural killer cells and increased the circulating serum levels of cytokines such as IFN-γ and IL-6 at early time points after the administration of the recombinant IL-15 agonist and nivolumab [40].

**Figure 6:**
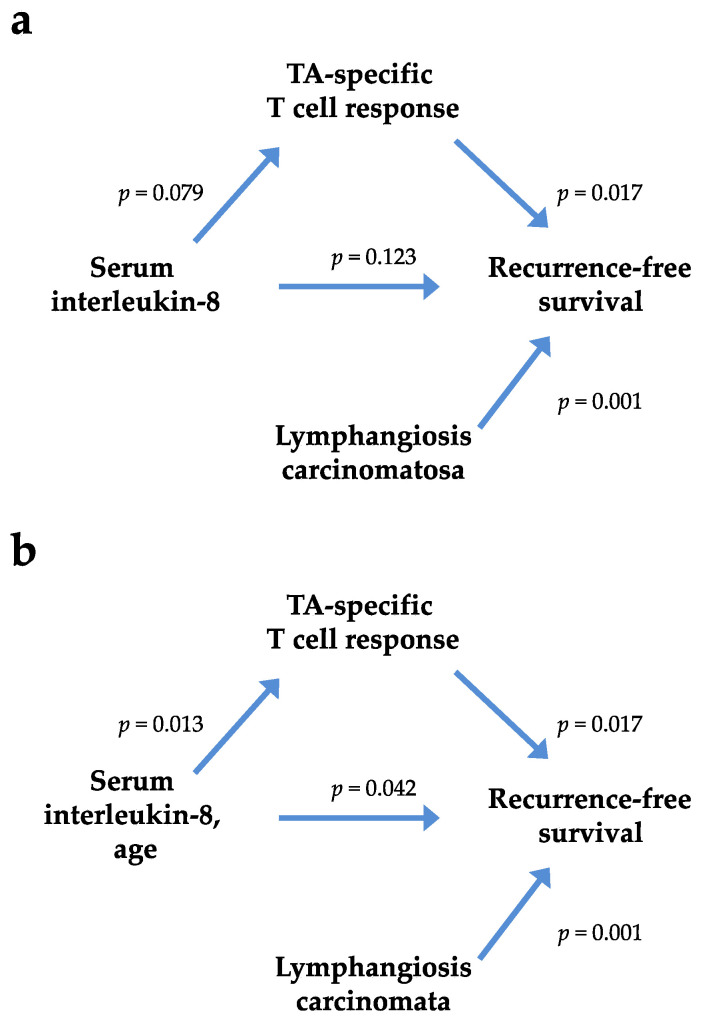
Associations among lymphangiosis carcinomatosa, the TA-specific T cell response, recurrence-free survival (RFS) and crude (**a**) and age-adjusted (**b**) serum IL-8 levels.

The postoperative tumor stage (Figure S6a), lymph node metastases (Figure S6b), and lymphangiosis carcinomatosa (Figure S6c) were decisive factors for tumor recurrence. A strong association was observed between serum IL-4 levels and TA-specific T cell responses (odds ratio (OR) = 11; *p* = 0.003; Table 2), as well as between TA-specific T cell responses and RFS (Figure S2a) and between lymphangiosis carcinomatosa and RFS (Figure S6c). IL-4 was associated with lymphangiosis carcinomatosa (*p* = 0.080; Table S7); however, in the logistic models of the TA-specific response, lymphangiosis carcinomatosa did not exert a direct effect on the variable response in either the crude or age-adjusted model. A significant age-adjusted hazard ratio for RFS was observed for IL-8, and a significant association was identified in the age-adjusted model for TA-specific T cell responses (Figure 6).

Unlike IL-4, we did not observe an association between IL-8 levels and lymphangiosis carcinomatosa (*p* = 0.511, Table S7). An imbalance between pro- and antiangiogenic factors in the tumor microenvironment may trigger structurally and functionally aberrant angiogenesis and lymphangiogenesis that facilitate tumor growth and metastasis. The relationship between IL-4 levels in the tumor microenvironment and an unproductive tumor vasculature remains unclear [41].

When the TA-specific T cell response was included as a variable in the multivariable Cox regression model for RFS, the effect of the serum IL-4 level disappeared due to the stronger effect of a TA-specific T cell response on RFS [6] and the strong correlation between the serum IL-4 level and the TA-specific T cell response. A potential explanation for this phenomenon is the inclusion of correlated predictors in multivariable regression analysis, which may lead to a significant collinearity effect and misleading interpretations of the results [42]. If the TA-specific T cell response was not available as a predictor, serum IL-4 levels would be the best single cytokine predictor of RFS. However, the data from this pilot study do not allow us to determine in which age subgroups certain cytokines are particularly associated with survival. The effect of cytokines on RFS (Table 4) remained after adjustment for age, indicating that, for example, the association between serum IL-4 levels and RFS is not explained by age.

This exploratory study has some limitations. First, we included both patients with squamous cell and non-squamous cell carcinomas, although these tumors are now recognized to have different molecular and immunological characteristics and clinical courses [43]. Second, validation in a larger cohort is needed to confirm the results of this pilot study. Third, due to the large number of comparisons, the resulting *p*-values were not adjusted for multiplicity and therefore are of a purely descriptive nature [44]. Despite these limitations, in this study, we comprehensively and simultaneously analyzed 27 different cytokines. This analysis distinguishes the approach used in our study from previous studies in which the levels of a single or a few cytokines were determined. We propose that a broader approach is more valid because clinical data from patients with cancer indicate the coexistence of cytokines that are associated with both immune stimulation and immunosuppression [9].

The occurrence of a clinically relevant immune response after checkpoint inhibitor therapy was largely based on pre-existing tumor-specific T cells in a study of patients with different types of advanced tumors [45]. The use of cytokines as monotherapies for cancer has not fulfilled expectations and is associated with toxicity at high doses [39]. However, cytokines might be useful not only in combination with established therapies to increase the activity of already existing TA-specific T cells and to promote tumor-specific immune responses [46] but also, according to the data in this study, as circulating biomarkers for preexisting TA-specific T cells in peripheral blood. 

## 4. Materials and Methods

### 4.1. Study Approval and Patients

The Ethics Committee in Heidelberg approved this prospective study (approval number: S-515/2013; ClinicalTrials.gov registration ID: NCT02515760). All investigations were performed in accordance with the principles outlined in the Declaration of Helsinki. This trial was conducted in the Department of Thoracic Surgery at Heidelberg University Hospital. The numbers of patients enrolled in the study and included in the analysis are outlined in the flow diagram shown in Figure Data S1.

### 4.2. Patient Samples

Fresh peripheral blood samples were obtained 1 day before surgery. Venous blood samples were subjected to Ficoll gradient (Biochrom, Berlin, Germany) centrifugation at 3000 rpm for 10 min, and cells in interphase were collected as previously described [47].

### 4.3. Preparation of Serum and Tumor Samples

Venous blood samples were centrifuged at 3000 rpm for 10 min. The serum was collected, aliquoted, and stored at −80 °C. A board-certified pathologist with expertise in NSCLC provided a macroscopically representative fresh tumor sample. Only tumor samples containing more than 40% tumor cells after hematoxylin–eosin staining and the pathological examination were selected for the multiplex analysis.

### 4.4. Surgery

All patients underwent general anesthesia and endobronchial double-lumen intubation. Surgery was performed via a video-assisted thoracoscopic approach or a thoracotomy approach with systematic nodal dissection in all patients with cancer [48,49]. A pathologist with expertise in lung cancer (WW) diagnosed the patients (Table 1). Lymphangiosis carcinomatosa was defined as pathologically confirmed peritumor invasion of lymphatic vessels by malignant cells [50].

### 4.5. Postoperative Follow-Up

The postoperative follow-up consisted of chest X-rays every three months and a chest computed tomography (CT) scan every six months. The occurrence of metastasis diagnosed during the first month after surgery was considered as stage IV undiagnosed at the time of surgery. This diagnosis led to the postoperative exclusion of patients with brain, bone, or pulmonary metastases from the final RFS analysis. Tumor recurrence was confirmed by the institutional tumor board. The primary end point was RFS, which was calculated from the time of tumor resection. Patients with high-risk UICC (7th edition) stage IB and stages II and higher were considered for adjuvant chemotherapy.

### 4.6. Cell Purification and Culture

Cell purification and culture for the IFN-γ ELISPOT and cytokine capture assays were conducted as previously described [47,51], Briefly, nonadherent peripheral blood mononuclear cells (PBMCs) were cultured for seven days in serum-free medium supplemented with 100 U/mL IL-2 plus 60 U/mL IL-4 and transferred into cytokine-free medium for 12 h before T cells were purified using a Dynabeads^®^ Untouched™ Human T Cells Kit (Thermo-Fisher, Schwerte, Germany). For dendritic cell (DC) generation, plastic-adherent mononuclear cells were cultured for seven days in serum-free medium supplemented with 50 ng/mL rhuGM-CSF (Essex Pharma, München, Germany) and 1000 U/mL IL-4 (PromoCell, Heidelberg, Germany). DCs were enriched using anti-CD19, anti-CD3, and anti-CD56-coupled Dynabeads^®^ Pan Mouse immunoglobulin G (IgG) (Thermo-Fisher). For antigen presentation, DCs were pulsed overnight with 200 μg of test antigens per 10^6^ cells/mL in cytokine-free X-VIVO 20 (Lonza, Köln, Germany).

### 4.7. Antigens

The Peptide Synthesis Facility of the German Cancer Research Center (DKFZ, Heidelberg, Germany) provided all polypeptides, which were designed to contain the known immunogenic HLA-A*0201 T cell epitope, as described previously in detail [6].

### 4.8. IFN-γ ELISPOT Assay

ELISPOT assays were performed as previously described [47], with a few modifications. Briefly, antigen-pulsed DCs were incubated with autologous T cells (DC:T cell ratio = 1:5) for 40 h in ELISPOT plates. The number of IFN-γ spot-forming cells was quantified using a CTL Analyzer (Cellular Technology, Cleveland, OH, USA). As a negative control, DCs were loaded with human IgG (Endobulin, Baxter, Unterschleissheim, Germany), which was considered a nonspecific background. Staphylococcal enterotoxin B (SEB) and cytomegalovirus (CMV) were used as positive controls. Individuals were considered responders if the spot numbers in triplicate test wells significantly (two-sided Student´s *t*-test with *p* < 0.05 as the responder criterion) exceeded the numbers in control wells.

### 4.9. Cytokine Quantification

The concentrations of 27 cytokines, chemokines, and growth factors in the serum samples and tumor lysates of patients with lung cancer were analyzed using multiplex technology (Bio-Rad Laboratories, Hercules, CA, USA) according to the manufacturer’s protocol. Briefly, proteins were quantified using a Pierce BCA protein assay kit (Thermo Scientific). Bioplex Manager software (version 6.1, Bio-Rad Laboratories) was used for data acquisition and analysis.

### 4.10. Statistical Analyses

For continuous variables, the median was reported as the location parameter of distribution. The Wilcoxon rank-sum test (Wilcoxon–Mann–Whitney test) was used to compare continuous variables between two groups, and Fisher´s exact test was used to evaluate the associations between two categorical variables. Spearman’s correlation coefficients and biserial correlations between binary and continuous variables [52] were calculated. The correlation matrix was constructed using a hierarchical clustering analysis (correlation distance metric and the ward linkage algorithm), and a dendrogram was created accordingly to visualize the associations between cytokine levels and the results from the IFN-γ ELISPOT assays. Logistic regression analyses were performed, and *p*-values from Wald chi-square and ORs with 95% confidence intervals (CIs) were reported for associations between the cytokine levels and the results of the IFN-γ ELISPOT assay. After adjustment for age, the adjusted ORs with 95% CIs were reported. Optimal cutoff values were determined by calculating the sensitivity and specificity to dichotomize the cytokine data for the logistic regression analysis. A stepwise forward selection approach (including the most significant variable, with *p* < 0.25 as the limit for inclusion and *p* < 0.10 as the limit for removal from the model) was used to determine the multiple logistic regression model that best predicted the results from the IFN-γ ELISPOT assays (presence versus absence of tumor-reactive T cells in the peripheral blood) using data for multiple cytokines.

For RFS, Kaplan–Meier curves were generated, and the results of the log rank test were reported. A Cox regression analysis was performed to determine the associations of RFS with the cytokine levels. The effects were indicated as the hazard ratio (HR) or as the adjusted hazard ratio (aHR) after adjustment for age groups.

All tests were two-sided, and *p*-values < 0.05 were considered statistically significant. Due to the exploratory nature of this analysis, none of the *p*-values were adjusted for multiple testing [44]. Statistical analyses were performed using the software Statistical Analysis System (SAS) for Windows, version 9.4 (SAS Institute Inc., Cary, NC, USA). All statistical analyses were supervised by a professional biostatistician.

## 5. Conclusions

Based on our data, preoperative serum IL-4 levels may have a role in predicting TA-specific T cell responses and RFS, regardless of the tumor stage. This finding is important because patients with high preoperative serum IL-4 levels are in a high-risk patient group for postoperative tumor recurrence and thus should be considered for adjuvant or neoadjuvant treatment. From this perspective, serum IL-4 measurements could be a useful option for all preoperative patients with NSCLC in the future. 

## Figures and Tables

**Table 1:** Nonparametric comparisons of the serum cytokine distributions in responders versus nonresponders and correlation coefficients between serum cytokine levels and the tumor-associated antigen (TA)-specific response.

Cytokine	*n*	Correlation Coefficients		Nonresponder (*n* = 14)	Responder (*n* = 22)	Wilcoxon Rank-Sum Test
		Rang Biserial	Spearman	Median	Median	*p*-Value
IFN-γ, serum	36	−0.0584	−0.0528	0.37	0.02	0.770
IL-10, serum	35	−0.1735	−0.1486	2.21	1.62	0.402
IL-1ra, serum	36	0.2890	0.2441	68.61	143.36	0.162
IL-4, serum	36	−0.4546	−0.3841	3.79	2.68	**0.030**
IL-5, serum	36	−0.1234	−0.1733	0.00	0.00	0.325
IL-6, serum	35	−0.0476	−0.0430	0.21	0.00	0.818
IL-7, serum	36	−0.2760	−0.2336	29.50	21.85	0.181
IL-8, serum	36	−0.2727	−0.2308	6.52	4.78	0.186
IL-9, serum	36	0.3507	0.2963	35.56	42.59	**0.091**
IL-12p70, serum	36	0.0390	0.0507	0.00	0.00	0.785
IL-13, serum	36	−0.2435	−0.2076	0.99	0.275	0.234
IL-15, serum	35	−0.1329	−0.1828	0.00	0.00	0.307
IL-17A, serum	36	−0.3571	−0.3019	21.79	14.96	**0.086**
Eotaxin, serum	36	−0.3961	−0.3346	74.13	56.15	**0.058**
Basic FGF, serum	36	−0.1786	−0.1549	7.38	3.39	0.374
G-CSF, serum	36	−0.4870	−0.4192	82.41	1.64	**0.019**
IP10, serum	36	−0.2857	−0.2413	439.00	328.28	0.167
MCP-1, serum	36	−0.0910	−0.0768	38.38	39.02	0.664
MIP-1a, serum	36	−0.1071	−0.0906	1.91	1.78	0.607
MIP-1b, serum	36	0.2727	0.2304	56.055	60.155	0.187
PDGF BB, serum	36	0.2987	0.2523	3386.99	4328.51	0.149
RANTES, serum	36	−0.0455	−0.0384	9543.66	9162.47	0.834
TNF-α, serum	36	0.0227	0.0192	13.805	14.15	0.923

Spearman’s rank correlation coefficients>|0.3288| are considered statistically significant for *n* = 36 patients. A responder was defined as a patient with T cells in the peripheral blood that were reactive to any of the 14 tested TAs in enzyme-linked immunospot (ELISPOT) analyses, and a nonresponder was defined as a patient without such T cells in the peripheral blood. Bold values indicate strong effects with a significant difference at *p* < 0.10. FGF = fibroblast growth factor. G-CSF = granulocyte colony-stimulating factor. IFN = interferon. IL = interleukin. IP10 = interferon-inducible protein 10. MCP-1 = monocyte chemoattractant protein 1. MIP = macrophage-inflammatory protein. PDGF = platelet-derived growth factor. RANTES = regulated upon activation, normal T cell expressed and secreted. TNF = tumor necrosis factor.

**Table 2:** Association between the TA-specific responses and the cytokine data. Results from crude and age-adjusted logistic regression models for responses according to the dichotomized cytokine data. Each cytokine was considered individually as a continuous variable and categorized using the optimal cutoff for the prediction of a TA-specific response. Odds ratios are provided for values below the respective optimal cutoffs (reference group “≥ cutoff” with OR = 1).

Variable	Cutoff (pg/mL)	Crude Logistic Regression Model		Model Adjusted for Age	
		OR (95% CI)	*p*-Value	aOR (95% CI)	*p*-Value
**IL-4, serum**	3.09	11.3 (2.3–55.0)	0.003	13.8 (2.3–82.1)	0.004
**IL-17A, serum**	21	4.5 (1.06–19.4)	0.042	6.4 (1.2–34.0)	0.029
**Eotaxin, serum**	58	5.3 (1.14–24.5)	0.033	5.9 (1.13–31.1)	0.036
**G_CSF, serum**	30	8.7 (1.6–48.5)	0.014	15.8 (2.0–122.3)	0.008
**IL-7, serum**	27	2.9 (0.71–11.4)	0.138	6.1 (1.03–36.1)	0.037
**IL-9, serum**	34.5	0.1 (0.012–0.6)	0.012	0.08 (0.011–0.57)	0.012
IL-1ra, serum	178	0.24 (0.04–1.35)	0.105	0.27 (0.05–1.63)	0.154
**IL-8, serum**	6	3.56 (0.86–14.63)	0.079	15.0 (1.78–126.5)	0.013
IP10, serum	400	4.38 (1.03–18.63)	0.046	3.69 (0.82–16.6)	0.089
MIP-1b, serum	58.5	0.23 (0.05–0.97)	0.046	0.26 (0.06–1.16)	0.077
PDGF BB, serum	4100	0.26 (0.06–1.07)	0.061	0.36 (0.08–1.67)	0.193

OR = odds ratio, aOR = adjusted odds ratio. Bold variables indicate significant effects (<0.05) in the age-adjusted model.

**Table 3:** Association between the TA-specific response and the cytokine data. Results from the multiple logistic regression model for the TA-specific response with the variables IL-4, IL-8, and MIP-1b (dichotomized) and age (continuous). Odds ratios are provided for values below the respective optimal cutoffs (reference group “≥ cutoff” with OR = 1).

Variable	Wald Chi-Square *p*-Value	Adjusted OR (95% CI)
Age [year]	0.0335	1.16 (1.00–1.35)
IL-4, serum (cutoff = 3.09 pg/mL)	0.0468	23.23 (1.70–318.1)
IL-8, serum (cutoff = 6 pg/mL)	0.0185	22.50 (1.12–453.3)
MIP-1b, serum (cutoff = 58.5 pg/mL)	0.0422	0.054 (0.003–0.89)

**Table 4:** Association between serum cytokine levels (dichotomized) and RFS. Log rank test of the survival analysis and HRs with 95% CIs from Cox regression models. Model 1 (M1): crude model; model 2 (M2): age-adjusted model; model 3 (M3): addition of the TA-specific response.

Variable	Log Rank	M1: Crude Cox Regression Model		M2: Age-Adjusted Cox Regression Model		M3: Cox Regression Model Including Cytokine Levels, the TA-Specific Response and Age			
		Cytokine		Cytokine		Cytokine		TA-Specific Response	
	*p*	HR (95% CI)	*p*	aHR (95% CI)	*p*	aHR (95% CI)	*p*	aHR (95% CI)	*p*
IL-4	**0.097**	2.8 (0.78–10.0)	**0.114**	2.8 (0.78–9.9)	**0.116**	1.4 (0.32–6.5)	0.634	0.30 (0.06–1.6)	**0.154**
IL-17A	0.175	2.3 (0.66–8.1)	0.190	2.2 (0.62–7.6)	0.222	1.3 (0.33–5.0)	0.724	0.27 (0.06–1.2)	**0.091**
Eotaxin	0.860	1.1 (0.32–4.0)	0.861	1.1 (0.30–3.8)	0.920	0.65 (0.17–2.6)	0.535	0.21 (0.05–0.93)	**0.040**
G CSF	**0.118**	3.2 (0.68–15.1)	**0.142**	3.3 (0.69–15.5)	**0.134**	2.0 (0.38–11.0	0.408	0.33 (0.07–1.4)	**0.138**
IL-7	0.482	2.1 (0.60–7.5)	0.243	2.6 (0.71–9.2)	0.152	1.8 (0.45–7.2)	0.407	0.30 (0.07–1.3)	**0.098**
IL-9	0.685	0.76 (0.20–2.9)	0.688	0.89 (0.23–3.5)	0.869	1.9 (0.44–8.1)	0.398	0.19 (0.04–0.83)	**0.028**
IL-1ra	0.908	0.92 (0.24–3.6)	0.908	0.97 (0.25–3.8)	0.969	1.8 (0.41–8.2)	0.432	0.20 (0.04–0.90)	**0.037**
IL-8	**0.106**	2.7 (0.76–9.6)	**0.123**	3.9 (1.05–14.5)	**0.042**	2.2 (0.50–10.1)	0.289	0.37 (0.07–1.9)	0.227
IP-10	0.446	1.6 (0.46–5.8)	0.454	1.2 (0.32–4.7)	0.760	1.0 (0.27–3.9)	0.971	0.25 (0.06-0.99)	**0.048**
MIP 1b	0.223	0.44 (0.11–1.7)	0.234	0.48 (0.12–1.9)	0.294	0.67 (0.16–2.8)	0.578	0.27 (0.07–1.1)	**0.072**
PDGF	0.712	1.3 (0.36–4.5)	0.715	1.7 (0.45–6.2)	0.438	2.8 (0.70–11.0)	**0.115**	0.18 (0.04–0.76)	**0.020**

Bold variables indicate strong effects with a significant difference at *p* < 0.15.

## Supplementary Materials

The following are available online at https://www.mdpi.com/2072-6694/12/12/3496/s1, Table S1: Characteristics of patients with (responders) and without circulating TA-specific T cells (nonresponders), Table S2: Characteristics of older and younger patients, Table S3: Bioplex data for cytokine levels measured in tumor tissues from 36 patients with NSCLC. Table S4: Bioplex data for cytokine levels measured in serum samples from 36 patients with NSCLC. Table S5: Association between median IFN-γ spot counts adjusted for the negative control values and the cytokine levels. Results from crude and age-adjusted linear regression models are shown. Table S6: Nonparametric comparisons of the intratumor cytokine distributions in responders and nonresponders and correlation coefficients between intratumor cytokine levels and the TA-specific response. Table S7: Association of dichotomized serum cytokine levels with potential risk factors or confounders. *p*-values are presented as the results of Fisher´s exact test. Figure S1: Consort diagram of the study. Figure S2: The number of IFN-γ spot counts significantly correlates with RFS. The median value of IFN-γ spot counts was calculated from the 36 patients. Survival based on IFN-γ spot counts above and below the median was correlated with response using two different spot count calculation methods: division of TA-specific spot counts by the corresponding IgG control counts (a) or subtraction of the IgG control counts from the corresponding TA-specific spot counts (b). Figure S3: Box and whisker plots presenting the tumor cytokine levels in all patients (maximum *n* = 36), responders only (*n* = 22) and nonresponders only (*n* = 14). Figure S4: Associations between median IFN-γ spot counts normalized to the values for the negative control and categorized serum cytokine levels. Figure S5: Correlations between serum levels of specific cytokines. Figure S6: Kaplan–Meier survival curves for postoperative tumor stage (a), lymph node status (b), lymphangiosis carcinomatosa (c) and preoperative dichotomized serum IL-4 levels (d). *p*-values represent the results of log-rank tests.

## References

[1] Ettinger D.S., Akerley W., Borghaei H., Chang A.C., Cheney R.T., Chirieac L.R., D’Amico T.A., Demmy T.L., Govindan R., Grannis F.W. (2013). Non-small cell lung cancer, version 2.2013. J. Natl. Compr. Canc. Netw..

[2] Siegel R.L., Miller K.D., Jemal A. (2017). Cancer statistics, 2017. CA Cancer J. Clin..

[3] Asamura H., Goya T., Koshiishi Y., Sohara Y., Eguchi K., Mori M., Nakanishi Y., Tsuchiya R., Shimokata K., Inoue H. (2008). A Japanese lung cancer registry study: Prognosis of 13,010 resected lung cancers. J. Thorac. Oncol..

[4] Yamauchi Y., Muley T., Safi S., Rieken S., Bischoff H., Kappes J., Warth A., Herth F.J., Dienemann H., Hoffmann H. (2015). The dynamic pattern of recurrence in curatively resected non-small cell lung cancer patients: Experiences at a single institution. Lung Cancer.

[5] Remark R., Becker C., Gomez J.E., Damotte D., Dieu-Nosjean M.C., Sautes-Fridman C., Fridman W.H., Powell C.A., Altorki N.K., Merad M. (2015). The non-small cell lung cancer immune contexture. A major determinant of tumor characteristics and patient outcome. Am. J. Respir. Crit. Care Med..

[6] Safi S., Yamauchi Y., Rathinasamy A., Stamova S., Eichhorn M., Warth A., Rauch G., Dienemann H., Hoffmann H., Beckhove P. (2017). Functional T cells targeting tumor-associated antigens are predictive for recurrence-free survival of patients with radically operated non-small cell lung cancer. Oncoimmunology.

[7] Grivennikov S.I., Greten F.R., Karin M. (2010). Immunity, inflammation, and cancer. Cell.

[8] Lee S., Margolin K. (2011). Cytokines in cancer immunotherapy. Cancers.

[9] Lippitz B.E. (2013). Cytokine patterns in patients with cancer: A systematic review. Lancet Oncol..

[10] Pine S.R., Mechanic L.E., Enewold L., Chaturvedi A.K., Katki H.A., Zheng Y.L., Bowman E.D., Engels E.A., Caporaso N.E., Harris C.C. (2011). Increased levels of circulating interleukin 6, interleukin 8, C-reactive protein, and risk of lung cancer. J. Natl. Cancer Inst..

[11] Sanmamed M.F., Perez-Gracia J.L., Schalper K.A., Fusco J.P., Gonzalez A., Rodriguez-Ruiz M.E., Onate C., Perez G., Alfaro C., Martin-Algarra S. (2017). Changes in serum interleukin-8 (IL-8) levels reflect and predict response to anti-PD-1 treatment in melanoma and non-small-cell lung cancer patients. Ann. Oncol..

[12] Margolin K., Morishima C., Velcheti V., Miller J.S., Lee S.M., Silk A.W., Holtan S.G., Lacroix A.M., Fling S.P., Kaiser J.C. (2018). Phase I trial of ALT-803, a novel recombinant IL15 complex, in patients with advanced solid tumors. Clin. Cancer Res..

[13] Miller J.S., Morishima C., McNeel D.G., Patel M.R., Kohrt H.E.K., Thompson J.A., Sondel P.M., Wakelee H.A., Disis M.L., Kaiser J.C. (2018). A first-in-human phase I study of subcutaneous outpatient recombinant human IL15 (rhIL15) in adults with advanced solid tumors. Clin. Cancer Res..

[14] Silva E.M., Mariano V.S., Pastrez P.R.A., Pinto M.C., Castro A.G., Syrjanen K.J., Longatto-Filho A. (2017). High systemic IL-6 is associated with worse prognosis in patients with non-small cell lung cancer. PLoS ONE.

[15] Barrera L., Montes-Servin E., Barrera A., Ramirez-Tirado L.A., Salinas-Parra F., Banales-Mendez J.L., Sandoval-Rios M., Arrieta O. (2015). Cytokine profile determined by data-mining analysis set into clusters of non-small-cell lung cancer patients according to prognosis. Ann. Oncol..

[16] Hayashi S., Imanishi R., Adachi M., Ikejima S., Nakata J., Morimoto S., Fujiki F., Nishida S., Tsuboi A., Hosen N. (2020). Reader-free ELISPOT assay for immuno-monitoring in peptide-based cancer vaccine immunotherapy. Biomed. Rep..

[17] Fane M., Weeraratna A.T. (2020). How the ageing microenvironment influences tumour progression. Nat. Rev. Cancer.

[18] Verschoor C.P., Johnstone J., Millar J., Dorrington M.G., Habibagahi M., Lelic A., Loeb M., Bramson J.L., Bowdish D.M. (2013). Blood CD33(+)HLA-DR(-) myeloid-derived suppressor cells are increased with age and a history of cancer. J. Leukoc. Biol..

[19] Rosenkranz D., Weyer S., Tolosa E., Gaenslen A., Berg D., Leyhe T., Gasser T., Stoltze L. (2007). Higher frequency of regulatory T cells in the elderly and increased suppressive activity in neurodegeneration. J. Neuroimmunol..

[20] Groth C., Hu X., Weber R., Fleming V., Altevogt P., Utikal J., Umansky V. (2019). Immunosuppression mediated by myeloid-derived suppressor cells (MDSCs) during tumour progression. Br. J. Cancer.

[21] Tanaka A., Sakaguchi S. (2019). Targeting Treg cells in cancer immunotherapy. Eur. J. Immunol..

[22] Wijesundara D.K., Tscharke D.C., Jackson R.J., Ranasinghe C. (2013). Reduced interleukin-4 receptor alpha expression on CD8+ T cells correlates with higher quality anti-viral immunity. PLoS ONE.

[23] Kienzle N., Olver S., Buttigieg K., Groves P., Janas M.L., Baz A., Kelso A. (2005). Progressive differentiation and commitment of CD8+ T cells to a poorly cytolytic CD8low phenotype in the presence of IL-4. J. Immunol..

[24] Kaminska J., Kowalska M., Kotowicz B., Fuksiewicz M., Glogowski M., Wojcik E., Chechlinska M., Steffen J. (2006). Pretreatment serum levels of cytokines and cytokine receptors in patients with non-small cell lung cancer, and correlations with clinicopathological features and prognosis. M-CSF—An independent prognostic factor. Oncology.

[25] Kim H.O., Kim H.S., Youn J.C., Shin E.C., Park S. (2011). Serum cytokine profiles in healthy young and elderly population assessed using multiplexed bead-based immunoassays. J. Transl. Med..

[26] Kleiner G., Marcuzzi A., Zanin V., Monasta L., Zauli G. (2013). Cytokine levels in the serum of healthy subjects. Mediat. Inflamm..

[27] Huang M., Wang J., Lee P., Sharma S., Mao J.T., Meissner H., Uyemura K., Modlin R., Wollman J., Dubinett S.M. (1995). Human non-small cell lung cancer cells express a type 2 cytokine pattern. Cancer Res..

[28] Yamauchi Y., Safi S., Blattner C., Rathinasamy A., Umansky L., Juenger S., Warth A., Eichhorn M., Muley T., Herth F.J.F. (2018). Circulating and tumor myeloid-derived suppressor cells in resectable non-small cell lung cancer. Am. J. Respir. Crit. Care Med..

[29] Aziz N. (2015). Measurement of circulating cytokines and immune-activation markers by multiplex technology in the clinical setting: What are we really measuring?. For. Immunopathol. Dis. Therap..

[30] Li Z., Jiang J., Wang Z., Zhang J., Xiao M., Wang C., Lu Y., Qin Z. (2008). Endogenous interleukin-4 promotes tumor development by increasing tumor cell resistance to apoptosis. Cancer Res..

[31] Guo N., Shen G., Zhang Y., Moustafa A.A., Ge D., You Z. (2019). Interleukin-17 promotes migration and invasion of human cancer cells through upregulation of MTA1 expression. Front. Oncol..

[32] Aliper A.M., Frieden-Korovkina V.P., Buzdin A., Roumiantsev S.A., Zhavoronkov A. (2014). A role for G-CSF and GM-CSF in nonmyeloid cancers. Cancer Med..

[33] Levina V., Nolen B.M., Marrangoni A.M., Cheng P., Marks J.R., Szczepanski M.J., Szajnik M.E., Gorelik E., Lokshin A.E. (2009). Role of eotaxin-1 signaling in ovarian cancer. Clin. Cancer Res..

[34] Yaseen M.M., Abuharfeil N.M., Darmani H., Daoud A. (2020). Mechanisms of immune suppression by myeloid-derived suppressor cells: The role of interleukin-10 as a key immunoregulatory cytokine. Open Biol..

[35] Nelms K., Keegan A.D., Zamorano J., Ryan J.J., Paul W.E. (1999). The IL-4 receptor: Signaling mechanisms and biologic functions. Annu. Rev. Immunol..

[36] Asselin-Paturel C., Echchakir H., Carayol G., Gay F., Opolon P., Grunenwald D., Chouaib S., Mami-Chouaib F. (1998). Quantitative analysis of Th1, Th2 and TGF-beta1 cytokine expression in tumor, TIL and PBL of non-small cell lung cancer patients. Int. J. Cancer.

[37] Ito S.E., Shirota H., Kasahara Y., Saijo K., Ishioka C. (2017). IL-4 blockade alters the tumor microenvironment and augments the response to cancer immunotherapy in a mouse model. Cancer Immunol. Immunother..

[38] Volonte A., Di Tomaso T., Spinelli M., Todaro M., Sanvito F., Albarello L., Bissolati M., Ghirardelli L., Orsenigo E., Ferrone S. (2014). Cancer-initiating cells from colorectal cancer patients escape from T cell-mediated immunosurveillance in vitro through membrane-bound IL-4. J. Immunol..

[39] Waldmann T.A. (2018). Cytokines in cancer immunotherapy. Cold Spring Harb. Perspect. Biol..

[40] Wrangle J.M., Velcheti V., Patel M.R., Garrett-Mayer E., Hill E.G., Ravenel J.G., Miller J.S., Farhad M., Anderton K., Lindsey K. (2018). ALT-803, an IL-15 superagonist, in combination with nivolumab in patients with metastatic non-small cell lung cancer: A non-randomised, open-label, phase 1b trial. Lancet Oncol..

[41] Baeriswyl V., Christofori G. (2009). The angiogenic switch in carcinogenesis. Semin. Cancer Biol..

[42] Vatcheva K.P., Lee M., McCormick J.B., Rahbar M.H. (2016). Multicollinearity in regression analyses conducted in epidemiologic studies. Epidemiology.

[43] Faruki H., Mayhew G.M., Serody J.S., Hayes D.N., Perou C.M., Lai-Goldman M. (2017). Lung adenocarcinoma and squamous cell carcinoma gene expression subtypes demonstrate significant differences in tumor immune landscape. J. Thorac. Oncol..

[44] Althouse A.D. (2016). Adjust for Multiple Comparisons? It’s Not That Simple. Ann. Thorac. Surg..

[45] Herbst R.S., Soria J.C., Kowanetz M., Fine G.D., Hamid O., Gordon M.S., Sosman J.A., McDermott D.F., Powderly J.D., Gettinger S.N. (2014). Predictive correlates of response to the anti-PD-L1 antibody MPDL3280A in cancer patients. Nature.

[46] Palucka A.K., Coussens L.M. (2016). The basis of oncoimmunology. Cell.

[47] Feuerer M., Beckhove P., Bai L., Solomayer E.F., Bastert G., Diel I.J., Pedain C., Oberniedermayr M., Schirrmacher V., Umansky V. (2001). Therapy of human tumors in NOD/SCID mice with patient-derived reactivated memory T cells from bone marrow. Nat. Med..

[48] Dienemann H., Hoffmann H., Koebe H.G. (1998). Technique and rationale of lymph node dissection in bronchial carcinoma. Chirurg.

[49] Lardinois D., De Leyn P., Van Schil P., Porta R.R., Waller D., Passlick B., Zielinski M., Lerut T., Weder W. (2006). ESTS guidelines for intraoperative lymph node staging in non-small cell lung cancer. Eur. J. Cardiothorac. Surg..

[50] Brierley J., Gospodarowicz M.K., Wittekind C. (2017). TNM Classification of Malignant Tumours.

[51] Reissfelder C., Timke C., Schmitz-Winnenthal H., Rahbari N.N., Koch M., Klug F., Roeder F., Edler L., Debus J., Buchler M.W. (2011). A randomized controlled trial to investigate the influence of low dose radiotherapy on immune stimulatory effects in liver metastases of colorectal cancer. BMC Cancer.

[52] Kraemer H.C., Kotz S., Johnson N.L. (1982). Encyclopaedia of statistical sciences. Biserial Correlation.

