# Supplementary material for: Circulating Interleukin-4 Is Associated with a Systemic T Cell Response against Tumor-Associated Antigens in Treatment-Naïve Patients with Resectable Non-Small-Cell Lung Cancer
Source: Cancers (Basel). 2020 Nov 24;12(12):3496. doi: 10.3390/cancers12123496 (PMC7761081; doi:10.3390/cancers12123496)
Supplement: Supplementary file 1 [file cancers-12-03496-s001.pdf]

Supplementary Materials: Circulating Interleukin-4 is Associated with a Systemic T Cell Response against Tumor-Associated Antigens in Treatment-Naïve Patients with Resectable Non-Small-Cell Lung Cancer

Seyer Safi, Yoshikane Yamauchi, Hans Hoffmann, Wilko Weichert, Philipp J. Jost, Hauke Winter, Thomas Muley and Philipp Beckhove

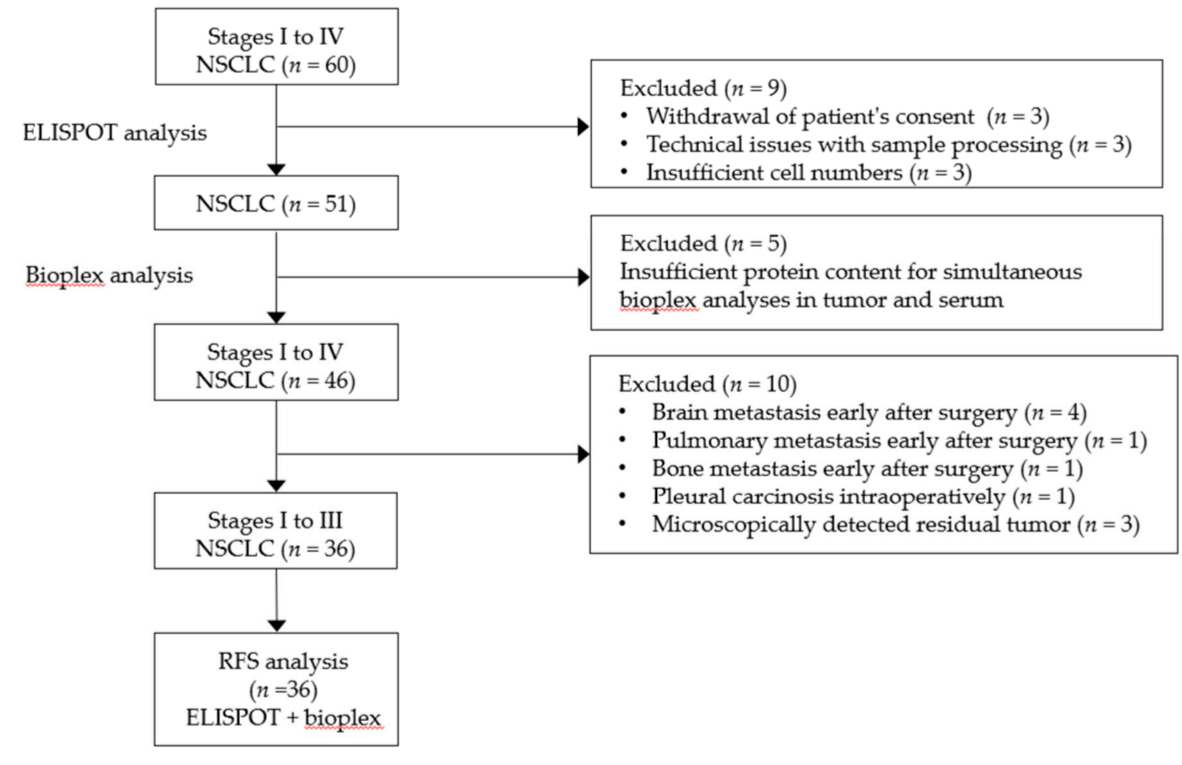

Figure S1. Consort diagram of the study.

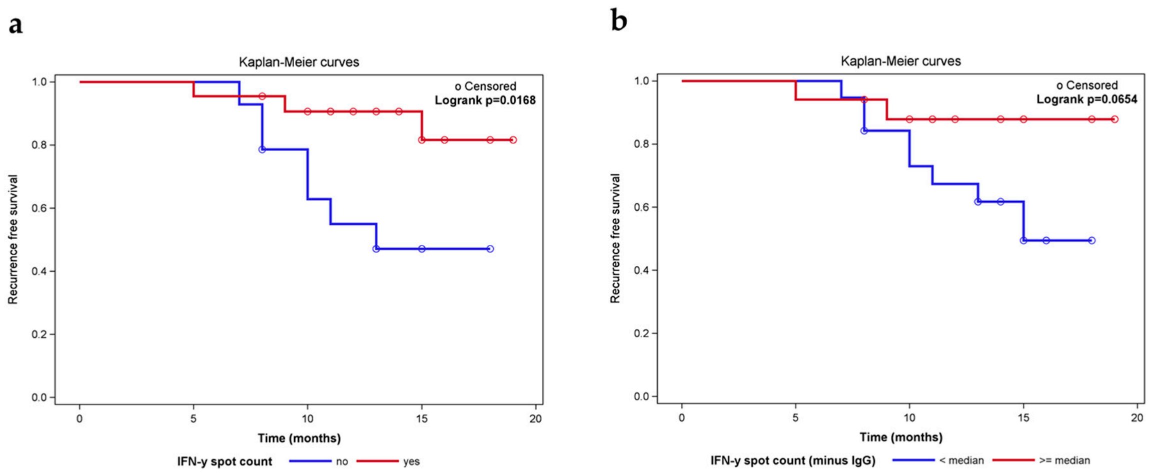

Figure S2. The number of IFN-γ spot counts significantly correlates with RFS. The median value of IFN-γ spot counts was calculated from the 36 patients. Survival based on IFN-γ spot counts above

and below the median was correlated with response using two different spot count calculation methods: division of TA-specific spot counts by the corresponding IgG control counts (**a**) or subtraction of the IgG control counts from the corresponding TA-specific spot counts (**b**).

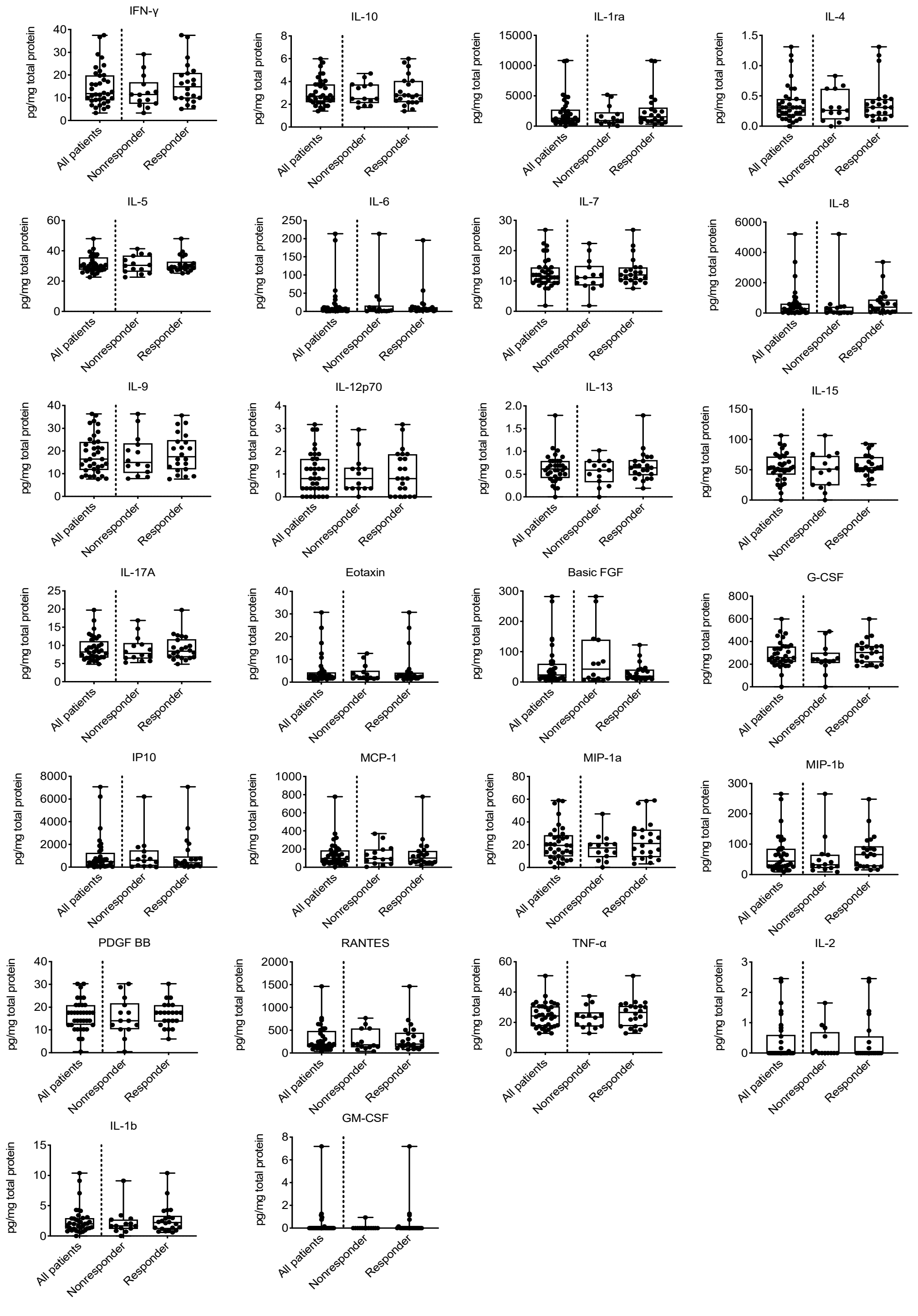

Figure S3. Box and whisker plots presenting the tumor cytokine levels in all patients (maximum n = 36), responders only (n = 22) and nonresponders only (n = 14).

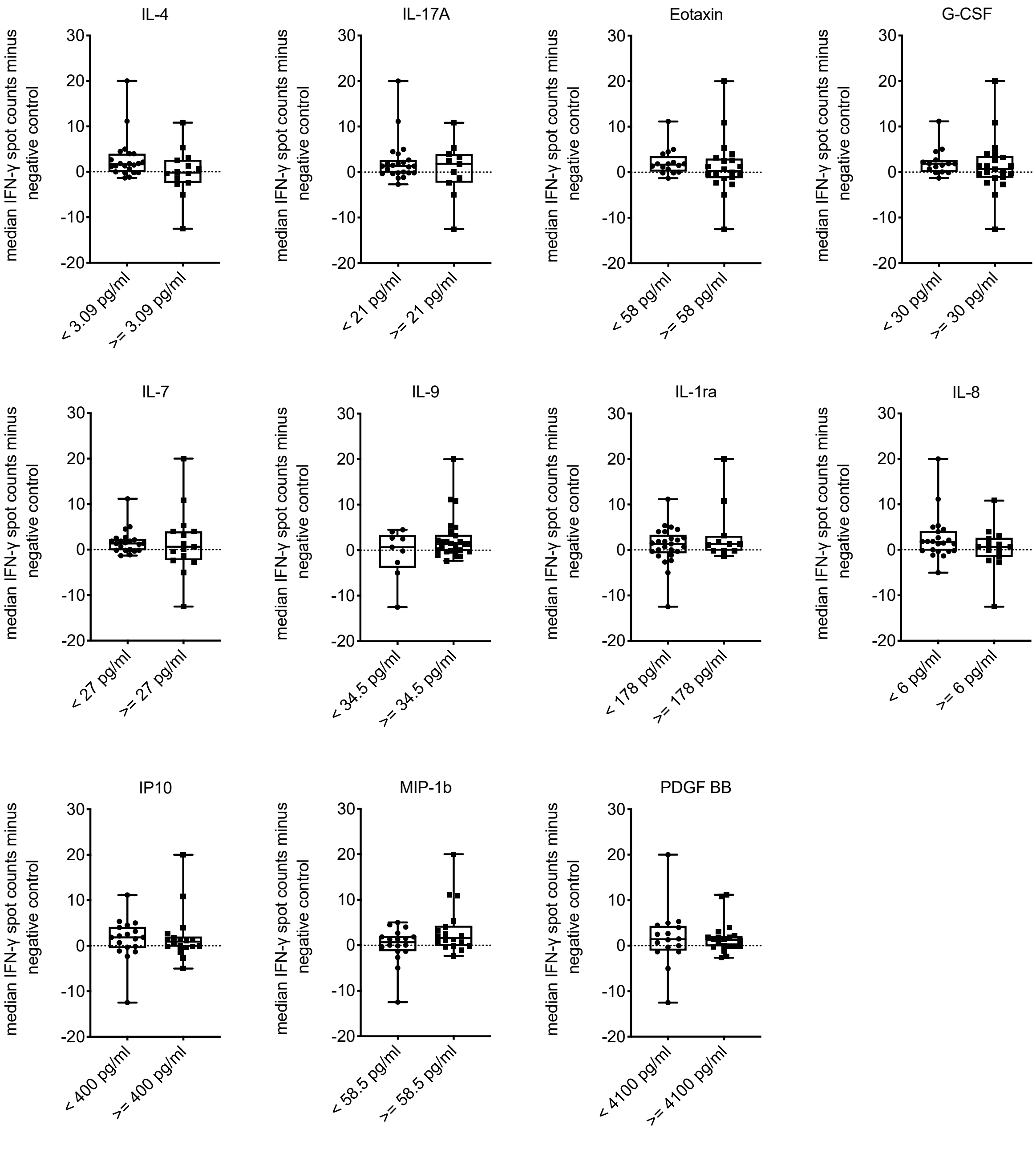

Figure S4. Associations between median IFN-γ spot counts normalized to the values for the negative control and categorized serum cytokine levels.

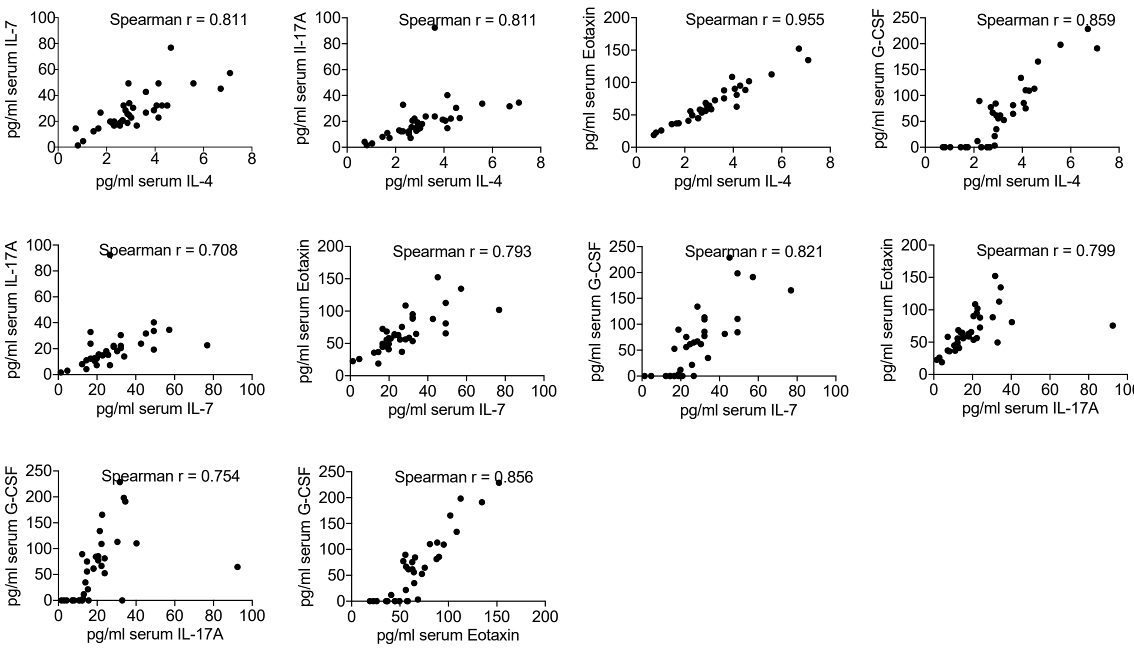

Figure S5. Correlations between serum levels of specific cytokines.

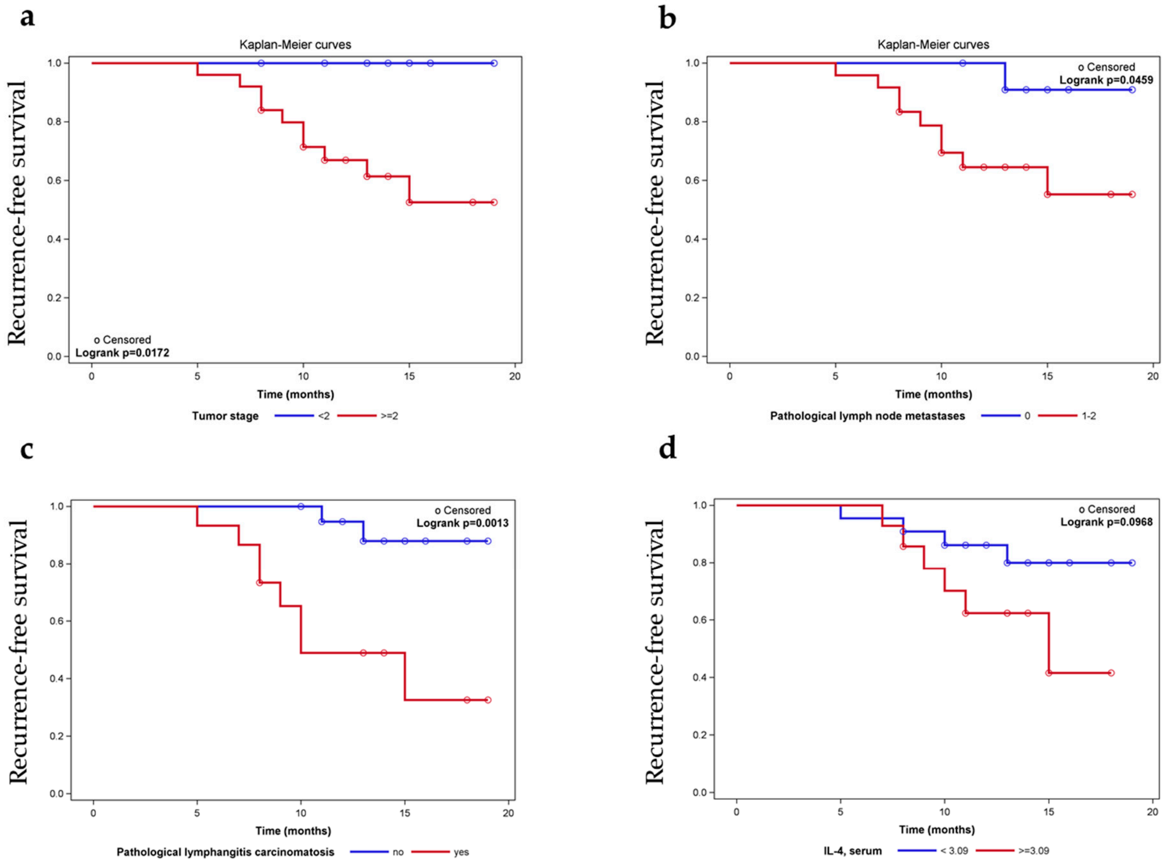

Figure S6. Kaplan–Meier survival curves for postoperative tumor stage (**a**), lymph node status (**b**), lymphangiosis carcinomatosa (**c**) and preoperative dichotomized serum IL-4 levels (**d**). *p*-values represent the results of log-rank tests.

Table S1. Characteristics of patients with (responders) and without (nonresponders) circulating TA-specific T cells.

Variable	T cell nonresponder *n* = 14	T cell responder *n* = 22	*p*-value *
Age, mean	70.0	63.9	**0.059**
Age ≥ 68 years, %	71.4	40.9	**0.097**
Male, %	42.9	50.0	0.742
Tumor stages II and III, %	71.4	68.2	1.000
Adjuvant chemotherapy given, %	42.9	50.0	0.742
Lymph node metastases present (pN1/2), %	71.4	63.6	0.727
Lymphangiosis carcinomatosa present (pL1) #, %	57.1	33.3	0.187

* Fisher's exact test/t-test; # *n* = 35; TA-specific = tumor antigen-specific. Bold values indicate strong effects with a significant difference at $p < 0.15$.

Table S2. Characteristics of older and younger patients.

Variable	All Patients *n* = 36	Patients <68 Years *n* = 17	Patients ≥68 Years *n* = 19	*p*-value *
Median age [years], (IQR)	68 (59 to 74)			
Male, %	47% (17)	53 (9)	42 (8)	0.739
Current or former smoker, %	83% (30)	94%	74%	0.182
Lobectomy for tumor resection, %	78% (28)	76% (13)	79% (15)	1.000
Median tumor size [mm], (IQR)	30 (21 to 42)	33 (22 to 42)	28.5 (21 to 39)	0.381
Pulmonary adenocarcinoma, %	64% (23)	76% (13)	53% (10)	0.177
Postoperative UICC tumor stage, 7th edition				0.913
I	30.5% (11)	35% (6)	26% (5)	
II	27.8% (10)	24% (4)	32% (6)	
III	41.7% (15)	41% (7)	42% (8)	
IV	0			
Lymph node metastases present (pN1/2), %	66.7% (24)	59% (10)	74% (14)	0.483
Lymphangiosis carcinomatosa present (pL1) #, %	42.9% (15)	29% (5)	56% (10)	0.176

* Fisher's exact test/Wilcoxon test; IQR = Interquartile range; # *n* = 35.

Table S3. Bioplex data for cytokine levels measured in tumor tissues from 36 patients with NSCLC.

Variable	*n*	Median	Mean	Min	Max	Comment
IFN-y, tumor	36	11.850	14.8297222	3.33	37.56	
IL-2, tumor	34	0	0.3750000	0	2.45	
IL-10, tumor	36	2.710	3.0552778	1.4	6.00	
IL-1b, tumor	36	1.995	2.5180556	0	10.37	
IL-1ra, tumor	36	1287.56	2224.54	33.8	10800.78	
IL-4, tumor	36	0.310	0.3758333	0	1.31	
IL-5, tumor	36	29.625	31.0300000	22.55	47.95	
IL-6, tumor	36	5.445	19.9475000	0	213.28	
IL-7, tumor	36	11.560	12.6602778	1.84	26.86	
IL-8, tumor	36	298.570	617.796667	4.87	5217.00	
IL-9, tumor	36	16.430	18.5375000	7.63	36.31	
IL_12p70, tumor	36	0.800	1.0350000	0	3.18	
IL-13, tumor	36	0.615	0.6283333	0	1.79	
IL-15, tumor	36	52.600	53.7963889	0	106.71	

IL17A, tumor	36	8.210	9.1169444	4.87	19.74	
Eotaxin, tumor	36	2.590	4.9183333	1.12	30.67	
Basic_FGF, tumor	36	22.810	49.5975000	4.34	281.93	
G-CSF, tumor	36	257.570	285.376111	0	598.25	
GM_CSF, tumor	35	0	0.3257143	0	7.18	
IP10, tumor	36	489.790	1018.18	4.41	7069.80	
MCP-1, tumor	36	96.975	140.116111	4.33	777.20	
MIP-1a, tumor	36	19.205	21.3177778	0.10	58.85	
MIP-1b, tumor	36	43.905	66.1322222	8.29	265.64	
PDGF_BB, tumor	36	17.580	16.3380556	0.46	30.26	
RANTES, tumor	36	192.805	304.706667	35.36	1462.04	
TNF-a, tumor	36	24.215	24.6386111	12.79	50.70	
VEGF, tumor	35	0	0	0	0	No analysis *

* Due to the large number of measurements out of range, this variable was excluded from analysis.

Table S4. Bioplex data for cytokine levels measured in serum samples from 36 patients with NSCLC.

Variable	*n*	Median	Mean	Min	Max	Comment
IFN-y, serum	36	0.020	33.6964	0	1115.20	
IL-2, serum	35	0	0.2923	0	6.32	No analysis *
IL-10, serum	35	1.910	4.3320	0	35.51	
IL-1b, serum	35	0	0.2054	0	6.42	No analysis *
IL-1ra, serum	36	122.825	141.0461	2.65	439.88	
IL-4, serum	36	2.880	3.12361	0.72	7.09	
IL-5, serum	36	0	6.9158333	0	181.18	
IL-6, serum	35	0.130	2.3891429	0	26.44	
IL-7, serum	36	25.270	27.9028	1.36	76.87	
IL-8, serum	36	5.430	493.9936	0	17533.00	
IL-9, serum	36	41.255	60.67250	17.02	421.14	
IL_12p70, serum	36	0	5.17222	0	99.94	
IL-13, serum	36	0.690	1.9081	0	22.82	
IL-15, serum	35	0	19.8594	0	291.46	
IL17A, serum	36	16.825	19.9481	1.59	92.42	
Eotaxin, serum	36	60.660	66.4169	18.89	152.19	
Basic_FGF, serum	36	3.390	12.9931	0	105.17	
G-CSF, serum	36	58.715	60.5144	0	228.63	
GM_CSF, serum	35	0	0.5489	0	14.51	No analysis *
IP10, serum	36	391.320	440.3514	158.29	1819.70	
MCP-1, serum	36	38.805	40.7219	4.16	144.14	
MIP-1a, serum	36	1.790	2.1783	0.33	5.69	
MIP-1b, serum	36	58.475	61.7344	37.02	111.69	

PDGF_BB, serum	36	4176.47	4138.54	1315.26	8051.01	
RANTES, serum	36	9162.47	9288.39	1175.35	15606.02	
TNF-a, serum	36	13.805	30.2406	1.83	263.96	
VEGF, serum	35	0	1.5963	0	55.87	No analysis *

* Due to the large number of measurements out of range, this variable was excluded from analysis.

Table S5. Association between median IFN-γ spot counts adjusted for the negative control values and the cytokine levels[1]. Results from crude and age-adjusted linear regression models are shown.

**Variable**	**Cutoff[1] (pg/mL)**	**Crude Linear Regression**		**Age-adjusted Linear Regression**	
		Effect[2]	*p*-value	Effect[2]	*p*-value
IL-4, serum	3.09	−2.87	**0.097**	−2.82	**0.108**
IL-17A, serum	21	−1.64	0.355	−1.66	0.357
Eotaxin, serum	58	−0.91	0.599	−0.84	0.631
G_CSF, serum	30	−1.06	0.539	−1.05	0.546
IL-7, serum	27	−0.04	0.980	−0.20	0.914
IL-9, serum	34.5	3.16	**0.104**	3.13	**0.112**
IL-1ra, serum	178	2.50	0.174	2.45	0.199
IL-8, serum	6	−2.16	0.214	−2.80	**0.138**
IP10, serum	400	0.54	0.755	0.70	0.692
MIP-1b, serum	58.5	3.33	**0.046**	3.32	**0.054**
PDGF BB, serum	4100	0.03	0.987	−0.29	0.881

[1] Optimal cutoff values calculated for logistic regression; [2] Difference in predicted means for the category "< cutoff" minus predicted means for the category ">= cutoff". Bold values indicate strong effects with a significant difference at $p < 0.15$.

Table S6. Nonparametric comparisons of the intratumor cytokine distributions in responders and nonresponders and correlation coefficients between intratumor cytokine levels and the TA-specific response.

Cytokine	*n*	Coefficients of Correlation		Nonresponder	Responder	Wilcoxon Rank-sum Test
		Rang biserial	Spearman	Median	Median	*p*-value
IFN-γ, tumor	36	0.2403	0.2029	11.41	14.85	0.244
IL-2, tumor	34	−0.0184	−0.0181	0.0	0.0	0.934
IL-10, tumor	36	0.0942	0.0798	2.48	2.79	0.651
IL-1b, tumor	36	0.1623	0.1371	1.66	2.21	0.432
IL-1ra, tumor	36	0.2078	0.1755	900.33	1537.01	0.314
IL-4, tumor	36	0.1526	0.1293	0.26	0.31	0.459
IL-5, tumor	36	−0.0065	−0.0055	30.33	29.28	0.987
IL-6, tumor	36	0.1916	0.1618	3.67	6.84	0.353
IL-7, tumor	36	0.2078	0.1762	11.13	11.99	0.312
IL-8, tumor	36	0.3507	0.2962	131.33	388.25	**0.091**
IL-9, tumor	36	0.1071	0.0905	14.90	17.44	0.607
IL-12p70, tumor	36	0.0130	0.0111	0.80	0.80	0.961
IL-13, tumor	36	0.1753	0.1487	0.59	0.64	0.394
IL-15, tumor	36	0.1916	0.1621	51.05	53.62	0.352
IL-17A, tumor	36	0.0812	0.0686	7.79	8.42	0.699
Eotaxin, tumor	36	0.0584	0.0494	2.18	2.79	0.784
Basic FGF, tumor	36	−0.1331	−0.1125	41.77	19.31	0.521
G-CSF, tumor	36	0.2662	0.2249	237.0	302.34	0.197
GM-CSF, tumor	36	0.1538	0.1961	0	0	0.272
IP10, tumor	35	−0.0584	−0.0494	609.75	384.50	0.784
MCP-1, tumor	36	−0.0065	−0.0055	96.98	102.32	0.987
MIP-1a, tumor	36	0.2273	0.1920	16.95	20.97	0.271
MIP-1b, tumor	36	0.2533	0.2139	31.93	65.93	0.220
PDGF BB, tumor	36	0.1331	0.1139	14.04	17.58	0.515
RANTES, tumor	36	0.0714	0.0603	180.53	196.02	0.735
TNF-α, tumor	36	0.1234	0.1043	23.43	26.57	0.552

Spearman rank correlation coefficients>|0.3288| are considered statistically significant for $n = 36$. A *responder* was defined as a patient with T cells in the peripheral blood that were reactive to any of the 14 tested tumor-associated antigens in ELISPOT analyses, and a *nonresponder* was defined as a patient without such T cells in the peripheral blood. Bold values indicate strong effects with a significant difference a $p < 0.10$.

Table S7. Association of dichotomized serum cytokine levels with potential risk factors or confounders. *p*-values are presented as the results of Fisher´s exact test.

Variables Dichotomized at the Optimal Cutoff	Age, Years <68 vs. ≥68	Stage I vs. ≥II	pL no vs. yes	Adj CTX no vs. yes	pN 0 vs. ≥1	Sex Male vs. Female
IL-4, serum	0.742	1.000	**0.080**	0.496	0.293	0.322
IL-17A, serum	1.000	0.708	0.157	0.299	0.468	0.177
Eotaxin, serum	1.000	0.718	**0.087**	0.749	0.730	0.749
G-CSF, serum	1.000	0.729	**0.037**	1.000	0.499	1.000
IL-7, serum	0.316	1.000	0.315	0.739	0.732	0.525
IL-9, serum	0.451	1.000	1.000	0.706	1.000	0.706
IL-1ra, serum	0.721	0.252	1.000	**0.003**	0.125	0.721

IL-8, serum	0.171	0.467	0.511	0.496	0.727	**0.097**
IP10, serum	**0.044**	1.000	0.738	0.181	0.725	1.000
MIP-1b, serum	0.505	0.471	1.000	1.000	0.725	1.000
PDGF BB, serum	**0.107**	0.718	0.506	0.335	0.482	0.335

Adj CTX = adjuvant chemotherapy, pL = lymphangiosis carcinomatosa, pN = lymph node metastases. Bold values indicate strong effects with significance <0.15.

Publisher's Note: MDPI stays neutral with regard to jurisdictional claims in published maps and institutional affiliations.

© 2020 by the authors. Licensee MDPI, Basel, Switzerland. This article is an open access article distributed under the terms and conditions of the Creative Commons Attribution (CC BY) license (http://creativecommons.org/licenses/by/4.0/).
